# Best Practices in Dengue Surveillance: A Report from the Asia-Pacific and Americas Dengue Prevention Boards

**DOI:** 10.1371/journal.pntd.0000890

**Published:** 2010-11-16

**Authors:** Mark E. Beatty, Amy Stone, David W. Fitzsimons, Jeffrey N. Hanna, Sai Kit Lam, Sirenda Vong, Maria G. Guzman, Jorge F. Mendez-Galvan, Scott B. Halstead, G. William Letson, Joel Kuritsky, Richard Mahoney, Harold S. Margolis, for The Asia-Pacific and Americas Dengue Prevention Boards Surveillance Working Group

**Affiliations:** 1 Pediatric Dengue Vaccine Initiative, International Vaccine Institute, Seoul, Republic of Korea; 2 Amy Stone Scientific and Medical Communications, Inc., Atlanta, Georgia, United States of America; 3 Department of Documentation and Communication, Office of Governing Bodies, Office of the Director-General, World Health Organization (WHO), Geneva, Switzerland; 4 The Asia-Pacific and Americas Dengue Prevention Boards Surveillance Working Group, Pediatric Dengue Vaccine Initiative, International Vaccine Institute, Seoul, Republic of Korea; Duke University-National University of Singapore, Singapore

## Abstract

**Background:**

Dengue fever is a virus infection that is spread by the *Aedes aegypti* mosquito and can cause severe disease especially in children. Dengue fever is a major problem in tropical and sub-tropical regions of the world.

**Methodology/Principal Findings:**

We invited dengue experts from around the world to attend meetings to discuss dengue surveillance. We reviewed literature, heard detailed reports on surveillance programs, and shared expert opinions.

**Results:**

Presentations by 22 countries were heard during the 2.5 day meetings. We describe the best methods of surveillance in general, the stakeholders in dengue surveillance, and the steps from mosquito bite to reporting of a dengue case to explore how best to carry out dengue surveillance. We also provide details and a comparison of the dengue surveillance programs by the presenting countries.

**Conclusions/Significance:**

The experts provided recommendations for achieving the best possible data from dengue surveillance accepting the realities of the real world (e.g., limited funding and staff). Their recommendations included: (1) Every dengue endemic country should make reporting of dengue cases to the government mandatory; (2) electronic reporting systems should be developed and used; (3) at minimum dengue surveillance data should include incidence, hospitalization rates, deaths by age group; (4) additional studies should be completed to check the sensitivity of the system; (5) laboratories should share expertise and data; (6) tests that identify dengue virus should be used in patients with fever for four days or less and antibody tests should be used after day 4 to diagnose dengue; and (7) early detection and prediction of dengue outbreaks should be goals for national surveillance systems.

## Author Summary

The Pediatric Dengue Vaccine Initiative organized Dengue Prevention Boards in the Asia-Pacific and the Americas regions consisting of dengue experts from endemic countries. Both Boards convened meetings to review issues in surveillance. Through presentations, facilitated discussions, and surveys, the Boards identified best practices in dengue surveillance including: (1) Dengue should be a notifiable disease in endemic countries; (2) World Health Organization regional case definitions should be consistently applied; (3) electronic reporting systems should be developed and used broadly to speed delivery of data to stakeholders; (4) minimum reporting should include incidence rates of dengue fever, dengue hemorrhagic fever, dengue shock syndrome, and dengue deaths, and hospitalization and mortality rates should be reported by age group; (5) periodic additional studies (e.g., capture/recapture) should be conducted to assess under-detection, under-reporting, and the quality of surveillance; (6) laboratory methods and protocols should be standardized; (7) national authorities should encourage laboratories to develop networks to share expertise and data; and (8) RT-PCR and virus isolation (and possibly detection of the NS1 protein) are the recommended methods for confirmation of an acute dengue infection, but are recommended only for the four days after onset of fever—after day 4, IgM-capture enzyme-linked immunosorbent assay is recommended.

## Introduction

Dengue virus, which is most commonly transmitted by the *Aedes aegypti* mosquito, is the most important mosquito-borne viral disease affecting humans [1]. Caused by one of four serotypes, dengue fever (DF) produces a spectrum of clinical illness that ranges from an influenza-like illness to a fatal shock syndrome (DSS). Most patients that progress to shock first develop a more severe form of infection called dengue hemorrhagic fever (DHF). We estimate that 3.6 billion people in 124 countries are at-risk for infection and 500 million people infected each year [2]. Over two million cases of DHF occur annually, and approximately 21,000 deaths are likely attributable to dengue [2].

Dengue prevention is limited vector control and treatment is limited to supportive care to avoid shock. To address the need for dengue prevention, several dengue vaccines are in development. One candidate entered expanded phase 2 clinical trials in 2009 [3].

Decision making prior to vaccine introduction and monitoring for effectiveness and safety after introduction require adequate country specific disease surveillance data [4]. To assess the status of dengue surveillance and to develop recommendation to improve surveillance data quality, two Dengue Prevention Boards convened to discuss dengue surveillance in representative countries. This report describes the results of that work.

## Methods

### Prevention Boards

As part of its program to facilitate the development and introduction of a dengue vaccine in endemic countries, Pediatric Dengue Vaccine Initiative (PDVI) [5] has sponsored two Boards consisting of dengue experts primarily from endemic countries, the Asia-Pacific Dengue Prevention Board (APDPB) and the Americas Dengue Prevention Board (AmDPB) [6]. These experts are in-country advocates for improved dengue prevention and control activities, most working in anticipation of dengue vaccines. The Boards meet regularly to assess various aspects of dengue prevention and control.

### Meetings on surveillance

Accurate burden of disease data will be needed for informed decision making regarding vaccine introduction [7]; however, often the only data available are from national surveillance. For this and other reasons, the Boards along with PDVI selected surveillance for their first topic to address. The format for their work was two working meetings of Board members and invited consultants and representatives from the Ministries of Health or other agencies involved in dengue surveillance. The objectives of the meetings were to assess the state of dengue surveillance in selected countries and reach a consensus on best practices. The Asia-Pacific Board met on June 19–21, 2007; the Americas Board, on January 17–19, 2008. In addition to Board members, meeting attendees included national and international experts in surveillance and dengue, representatives of ministries of health, WHO and regional offices (e.g. SEARO), PAHO and the Caribbean Epidemiology Center (CAREC). Oral presentations, facilitated discussions, and a survey of presenters were used to determine the key issues and best practices.

**Figure 1:**
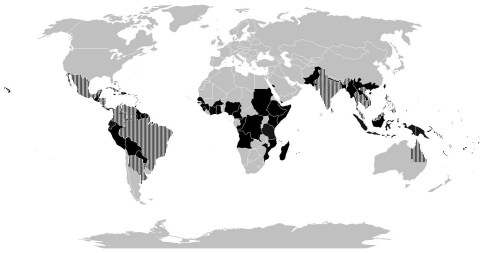
Countries with local dengue transmission in the last 25 years [**2**].

In total, there were presentations on the surveillance programs from twenty two countries given by representatives of Ministry of Health or other agency participating in dengue surveillance in-country (e.g. Institute Pasteur) (for APDPB: Australia, Cambodia, French Polynesia, India, Malaysia, the Philippines, Sri Lanka, Singapore, Thailand, Japan, Vietnam; for AmDPB: Argentina, Brazil, Costa Rico, Colombia, Cuba, Honduras, Mexico, Puerto Rico, Nicaragua, United States (South-west border states), and Venezuela) (Figure 1). Because ensuring adequate surveillance requires participation from several disciplines, experts presented on topics of surveillance, epidemiology, entomology, and virology. Each country provided a detailed description of their national dengue surveillance system and results (Table 1 & 2). Attendees then synthesized the comments and opinions of the Board members. Full reports of each meeting are be available on the website of the Prevention Boards [6].

**Table 1:** Characteristics of national surveillance systems.

Country	Case Definition*	Surveillance Method†	Source of Cases‡	Ages	Reporting Legally Required§	Vector Surveillance Conducted¶
Queensland, Australia	WPRO	Passive	OP, IP	All	Yes	Yes
Cambodia	WPRO	Passive	IP	0–15	Yes	No
		Sentinel	IP	0–15		
French Polynesia	WPRO	Passive	OP, IP	All	Yes	No
India	SEARO	Sentinel	OP, IP	All	No	Yes
Japan	WPRO	Passive	OP, IP	All	No	No
Malaysia	WPRO	Passive	OP, IP	All	Yes	Yes
Philippines	WPRO	Passive	OP,IP	All	Yes	No
		Sentinel	IP	All		
Singapore	WPRO	Passive	OP, IP	All	Yes	Yes
Sri Lanka	SEARO	Passive	OP, IP	All	Yes	Yes
Thailand	SEARO	Passive	IP	All	Yes	No
		Sentinel	IP	All		
Vietnam	WPRO	Passive	OP, IP	All	Yes	Yes
		Sentinel	OP, IP	All		
Argentina	PAHO	Passive	OP, IP	All	Yes	Yes
Brazil	PAHO	Passive	OP, IP	All	Yes	Yes
Colombia	PAHO	Passive	OP, IP	All	Yes	No
Costa Rica	PAHO	Passive	OP, IP	All	Yes	Yes
Cuba	PAHO	Active	OP, IP	All	Yes	Yes
Honduras	PAHO	Passive	OP, IP	All	Yes	Yes
Mexico	PAHO	Passive	OP, IP	All	Yes	Yes
Nicaragua	PAHO	Passive	IP	All	Yes	No
Puerto Rico	PAHO	Passive	OP, IP	All	Yes	No
United States	PAHO	Passive	OP, IP	All	No	No
Venezuela	PAHO	Passive	OP, IP	All	Yes	No

*Case definition used for dengue surveillance was same as World Health Organization Region Office recommended definition: WPRO  =  Western Pacific Regional Office; SEARO  =  Southeast Asia Regional Office; Pan American Health Organization;

**†:** Method of case ascertainment by national dengue surveillance system: active, passive or sentinel site surveillance;

**‡:** Source or location where cases are detected: OP  =  Outpatient clinics; IP  =  Inpatient or hospitalized;

**§:** Reporting of dengue cases is mandated by law;

**¶:** Mosquito surveillance is included as part of the national surveillance system and is not just in response to outbreaks.

**Table 2:** Dengue surveillance data from countries represented at the Prevention Board Meetings.

Country	Population	Total Cases (Year)	Reported DF∶DHF	DHF Case Fatality Rate	Peak Age (years)	Website for current dengue surveillance data
Asia-Pacific Region						
Australia(North Queensland)	664,440	58 (2007)140 (2008)	No DHF reported	ND*	32	http://www.health.qld.gov.au/dengue
Cambodia	13,000,000	9,040 (2005)16,649 (2006)	1.7∶1	0.9%	5–10	http://www.moh.gov.kh/
French Polynesia	132,900	3 (2005)818 (2006)	333∶1	<0.1%	10–19	http://www.vice-presidence.gov.pf/UserFiles/Bull%20Aout%2008.pdf
India	1,028,000,000	11,985 (2005)9,680 (2006)	ND	ND	14–45	http://www.nvbdcp.gov.in/
Japan	Travelers Only	31 (2003)49 (2004)	ND	ND	N/D	http://www.nih.go.jp/vir1/NVL/DengueNet%20Web/egdenguejp.htm
Malaysia	24821286	39,654 (2005)18,240 (2006)	16∶1	3.6%	20–24	http://www.moh.gov.my/pr_categories/1/press_releases
Philippines	84,241,341	33,490 (2005)36,891 (2006)	2∶3	<1%	1–10	http://www2.doh.gov.ph/nec
Singapore	4553009	14,209 (2005)3,100 (2006)	35∶1	0.003	15–24	http://www.dengue.gov.sg/
Sri Lanka	20926315	5,965 (2005)11,972 (2006)	3∶1	0.4%	20–29	http://www.epid.gov.lk/
Thailand	62,000,000	34,291 (2005)	10∶1	<0.2%	10–15	http://epid.moph.go.th
Vietnam	83,119,916	60,982 (2005)77,818 (2006)	ND	<0.1%	<15	http://www.wpro.who.int/health_topics/dengue/data.htm
Americas Region						
Argentina	38,592,150	34 (2005)181 (2006)	ND	ND	ND	http://www.paho.org/english/ad/dpc/cd/dengue.htm
Brazil	189,335,187	203,789 (2005) 346,550 (2006)	551∶1	11%	20–39	http://portal.saude.gov.br/SAUDE/area.cfm?id_area=920 (Boletim)
Colombia	42,090,512	39,825 (2005)38,271 (2006)	6∶1	5%	ND	http://www.bvs-vspcol.bvsalud.org/php/index.php (Notificación Semanal Obligatoria)
Costa Rica	4.401.845	37,798 (2005)12,052 (2006)	137∶1	ND	10–34	http://www.ministeriodesalud.go.cr/index.php/inicio-vigilancia-salud-boletines-ms
Cuba	11,416,987	14,8883(2001–2002)	178∶1	4%	adults	http://www.paho.org/english/ad/dpc/cd/dengue.htm
Honduras	7,400,000	18843 (2005)7800 (2006)	45∶174∶1	3%	≥15	http://www.paho.org/english/ad/dpc/cd/dengue.htm
Mexico	108700891	29,836 (2006)16,862 (2005)	4.8∶1	0.4%	10–14	http://www.dgepi.salud.gob.mx
Nicaragua	5,142,098	13,831 (2005)10,073 (2006)	26∶1	2%	5–9	http://www.minsa.gob.ni/vigepi/html/boletin.html
Puerto Rico	3,937,316	6,039 (2005)3,286 (2006)	84∶1	6%	15–19	http://www.salud.gov.pr/Datos/VDengue/Pages/default.aspx (Spanish)http://www.cdc.gov/ncidod/dvbid/dengue/documents/Weeklyreport.pdf (English)
United States†	11,000,000	28 (2005)	16∶9	0	ND	http://www.cdc.gov/dengue/epidemiology/index.html#surv
Venezuela	26084662	39,860 (2006)42,198 (2005)	9∶1	0.1%	2–9	http://www.mpps.gob.ve/modules.php?name=Downloads&cid=31

*ND  =  No data;

**†:** USA-Mexico border only.

Additional websites with current dengue data: PAHO: http://www.paho.org/english/ad/dpc/cd/dengue.htm; Asian ArboNet: http://www.nih.go.jp/vir1/NVL/DengueNet%20Web/ToppageArboNet.htm; Caribbean Epidemiology Centre: http://www.carec.org/; WHO: http://www.who.int/globalatlas/default.asp.

## Results

### Observations on surveillance systems

The core functions of a comprehensive surveillance system are detection, reporting, investigation, confirmation, analysis, interpretation, and response. Cooperation is essential between the healthcare system and the public health authority because for rapid response to emerging public health threats the public health authority is dependent on healthcare system to generate timely and accurate case reports.

### Observations on diagnosis and case definitions of dengue

At the time of the meeting, WHO had published guidelines on the diagnosis of dengue including case definitions; but these guidelines were published more than 10 years ago [8]—in 2009 WHO published new guidelines with major changes in dengue case classifications [9]. Regional offices have also drafted guidelines [10]–[12]. The guidelines agree on major issues with minor variations (for example, some include leukopenia or hepatomegaly in the case definitions, but not all include a “suspected case” category).

One major difficulty with all previous guidelines is case classification [13]. Because case fatality rates are much higher among patients with DHF, correct classification is important for triage, treatment, and prognosis. Obtaining a platelet count, hematocrit, and radiographic imaging is often not possible, too time consuming, or too expensive in many healthcare facilities in endemic countries—but the results of these tests are required diagnostic criteria for DHF. There was wide recognition of the need for a simplified classification system that is still helpful for case management [13], [14].

Although meeting attendees reported using similar dengue case definition systems, surveillance methods varied between countries. Laboratory methods also vary as well as the testing algorithms and the interpretation of positives. For example, in Brazil and Colombia, healthcare providers complete case reports on both ambulatory and hospitalized patients, however, in Thailand and Vietnam the majority of reported cases are hospitalized. In only 12/22 (55%) of countries represented at the meeting confirmed all officially reported cases with laboratory testing. Nearly every country includes suspected dengue cases regardless of age, but in Cambodia surveillance is conducted only among children less than 15 years of age. In Singapore and Brazil, monitoring vector indices is an integral part of the dengue surveillance system, while in Puerto Rico it is not. The attendees reported that these differences were not currently a problem for country level analyses but make inter-country, regional, and global analyses and comparisons difficult. Moreover, some difference (e.g. lack of dengue surveillance among adults in Cambodia) could be an impediment to strategic planning and implementation of a dengue vaccine since the disease also affects adults as well as children. Moreover, the vaccination of children is likely to also have an impact on adult disease burden [15], [16], further improving the cost-effectiveness.

**Figure 2:**
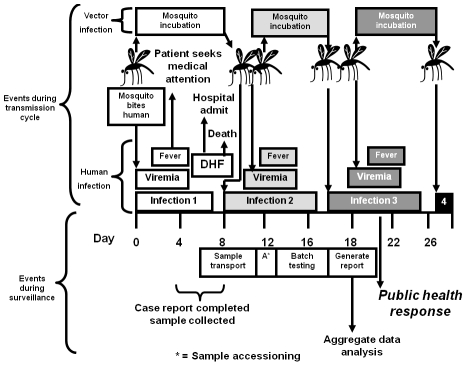
Timeline showing transmission cycle, clinical disease, and surveillance events. After the first infection results in clinical disease several additional infections occur before a public health response occurs in response to the index case.

Since surveillance data are needed for health ministries to target control responses when outbreaks are detected, data must be collected in a timely fashion. In order to better understand the overall process, attendees reviewed the steps from infection to reporting (Figure 2). The incubation period is, on average, one week following the bite of an infectious mosquito. Several more days pass before symptoms become severe enough to cause the patient to seek medical attention, and still more time is required for the symptoms of DHF to develop. Outpatient clinic-based surveillance will detect cases earlier than inpatient facilities, potentially allowing more time for public health action.

The medium for reporting ranged from paper case report forms, to hand-held computers, to internet-based systems. A case study from Nicaragua showed that hand-held computers, although initially requiring significant investment in infrastructure and training, do reduce reporting time. In Kolkata, India, special mapping of cases has been used to target control activities. In Singapore and Brazil, ministries are also using intranet-based data entry software allowing staff to directly enter data on cases and *Ae. aegypti* breeding sites in the field. The data are then immediately available to plan interventions and follow-up. All countries are dependent on paper forms for case reporting before any additional investigation or action. Time is required for that report to reach the surveillance office, to be entered and analyzed, and finally be reported. However, many countries are developing improved methods for data collection for targeted interventions.

Another key issue is the needs of stakeholders with interests in dengue diagnosis and surveillance. These stakeholders include the general public, senior policy-makers, academics, and legislators. A diverse group, their interests range from the parents of sick children who want immediate and accurate test results—knowing the diagnosis allows them to cope better—to healthcare workers, staff in laboratories, public health and vector control authorities. All want a point-of-care test to speed accurate diagnosis and treatment and allow rapid public health intervention. Others (e.g. general public, including travellers and Ministries of Health) are more likely interested in more accurate tests to allow improved burden of disease estimates which could effect budget allocations for control.

In most countries diagnostic testing and surveillance relies on healthcare practitioners and laboratory staff to report cases but they receive little benefit. Confirmatory diagnostic tests such as virus isolation or reverse transcriptase-polymerase chain reaction testing (RT-PCR) require expertise and equipment usually found only in reference laboratories. However, several attendees explained that the time required for a sample to reach and to be processed at centralized facilities often results in delays that render the results useless to the treating physician. Further delays occur if the information provide on a sample is incomplete or if batch-testing of samples is conducted. After testing, the report generated requires verification, approval and delivery (e.g. mailing). As a consequence, health care providers in most countries must treat patients empirically [17].

The attendees concluded that simplified case reporting [18], rapid turnaround of results, and training healthcare providers in reporting [19] can be important ways to encourage continued reporting of cases. Mandatory reporting, they explained, rarely guaranteed reporting.

### Strengths and weaknesses of existing surveillance systems

#### Strengths

Attendees were asked to identify the strengths and weaknesses of their systems. Most indicated that their countries had adequate infrastructure and surveillance systems, and the adjectives “dedicated”, “committed”, “skilled”, and “motivated” were widely used to describe the quality of the personnel engaged in surveillance. They reported some country-specific but effective links between the various stakeholders; especially healthcare providers, laboratory staff, and the public health and vector control authorities. However, many of these relationships are dependent on personal contacts which are affected by staff turnover.

#### Weaknesses

A common perception among meeting attendees was that disease control is politically more important than prevention. That is, highly visible outbreak response through spraying is considered more important than outbreak prevention. Specifically, during outbreaks, public demand for action often leads to pesticide spraying [20]–[23] which is unlikely to be effective since the pesticide released in the streets is unlikely to reach the adult mosquitoes resting and feeding inside homes [24].

Lack of preventive services in the provinces is seen as a further impediment to conducting adequate surveillance. Further, even when adequate infrastructure exists, data are rarely used locally; rather they are forwarded to the central ministry offices for official evaluation, missing the opportunity for an immediate local response. Diagnostic tests further complicate the situation because the results are often difficult to interpret by the healthcare providers and public health practitioners unfamiliar with the limitations of the tests [25]. Lack of funding for laboratory confirmation of cases and having those services available only at central level were reported as further weaknesses. One participant remarked that local pubic health agencies in large countries such as Brazil have their response time greatly delayed if they must wait for laboratory confirmation at the national level. Indeed, while experts agreed that staff conducting surveillance were committed, under-detection and under-reporting of dengue cases were significant and often due to the design of the surveillance system and lack of funding. Also, data sharing and full coordination of entomologic surveillance conducted by vector control units and human disease surveillance conducted by epidemiologists is needed to improve detection of increased transmission sufficiently early to prevent or control outbreaks. Finally, virological surveillance is under-utilized or in some countries, completely lacking: It's importance emphasized by the fact that large outbreaks tend to follow changes or reintroductions of serotypes.

## Discussion

As an outcome of the meeting, attendees agreed on best practices on laboratory practices, data gathering, analyses, reporting, and feedback for dengue surveillance.

### Guiding principles

Every dengue endemic country should systematically gather data in an established dengue surveillance system [12], and each system should have a quality assurance mechanism. Legislation should make dengue a notifiable disease in every affected country [12] to improve the capture of cases by surveillance. However, even mandatory reporting is not sufficient; additional efforts are needed to improve and maintain a high level of quality reporting. All suspected cases must be reported to a central dengue unit in the health ministry as rapidly as possible and providers should be reminded that timely reporting can lead to effective response [26]. Laboratory confirmation of suspected cases should always be sought, except during outbreaks. Once an outbreak is confirmed no added information is gained by testing all samples; a subset of the samples is usually sufficient to track the outbreak [17]. That said, health providers should be informed that not all samples submitted during outbreaks will necessarily be tested. In outbreaks, data collection and analysis should be completed as rapidly as possible.

Reporting should be encouraged from all levels of healthcare facilities in both the public and the private sectors [12]. In particular, mechanisms to involve the private sector should be developed; one possible way to encourage reporting is a rapid turn around of dengue diagnostic test results which can be provided free of charge[12]. While the turn around may not be quick enough to affect patient care, rapid return of results to submitting providers has intrinsic value for improving their diagnostic acumen. Reporting should be expanded to also include cases presenting to outpatient facilities, but staff in such settings may need further training to ensure the quality of data. To confirm and understand the burden of disease, periodic additional studies (e.g. using capture-recapture methods) should be conducted and incorporated into the system when possible. This will also determine the representativeness of the surveillance data.

### Laboratory practices

Laboratory confirmation improves the specificity of surveillance [27], but laboratory methods and protocols should be standardized. This can be achieved through national and international networking of dengue laboratories to share expertise, protocols and data.

A critical element for the successful laboratory diagnosis of an acute dengue infection is the timely collection of high quality samples. Monitoring the time from case identification to receipt of blood samples in the testing facility may assist in maintaining high quality specimens.

RT-PCR and virus isolation are the two recommended methods for virus identification. Monitoring serotypes and sequencing isolates can provide useful markers for outbreak prediction [28]. Detection of the non-structural protein antigen NS1 may also be useful, but it must undergoing further evaluation [17]. For serologic testing, the hemagglutination-inhibition assay remains the gold standard of serological assays and should be maintained in those laboratories capable of performing it; however, enzyme-linked immunosorbent assays for IgM and IgG are considered the minimum requirement for confirmation of cases [29].

#### Testing schedules

In the first four days after onset of fever, either RT-PCR or virus isolation are the recommended assays for confirmation of dengue infection [30]. IgM antibody detection is an alternative if virus detection or isolation is negative, but may not be detectable in the early stages of the illness [31]. After day 4, serology is the method of choice [27]. Paired blood samples collected on days 0–4 of illness and days 10–21 of illness are necessary for definitive serological diagnosis by IgM seroconversion. Where possible, IgG antibody detection should also be performed, particularly on suspected secondary dengue infections due to the absence of IgM antibody in up to 30% of those cases [32]. A four-fold increase in titer of IgG is also consistent with a recent dengue infection, but IgG antibodies are not dengue-specific and may have been caused by flaviviruses other than dengue [31].

#### Quality assurance and control

Support should be provided for quality control, proficiency testing and good laboratory practice at the WHO reference laboratory level, the national level and the local level [17]. Every assay should include standards (i.e. positive, negative and cut-offs). Experience and methodologies should be shared. Financial support for reagent production and distribution to national laboratories would reduce some variability in results and reduce cost. In the past, several WHO reference laboratories provided reagents free of charge on request to national laboratories. This improved comparability of results but was not financially sustainable.

### Reporting

At least weekly reporting of aggregate results was considered by the attendees as the minimum standard during peak transmission. To conserve resources, reporting could be reduced to biweekly during periods of low transmission. During an outbreak, more frequent reporting, perhaps on a daily basis, would be useful. However, it is important to note that reporting would be affected by the operating hours of the reporting facilities (e.g. facilities closed on weekends or holidays could artificially reduce reported cases and create surveillance artifacts). Reports should reach the surveillance units within 48 hours of form completion.

Especially since dengue occurs frequently in young adults in Southeast Asia, it is recommended that the usual categories for reporting in health information systems should be used, namely less than 1 year, 1–4 years, 5–14 years and older than 15 years. However, reporting the median age of cases across all ages is also a useful statistic to track, and may be more useful for comparison if countries are using different age categories. Moreover, if the median age is reported by countries not including cases of all ages because the peak incidence is in children, the overall age distribution could be modeled with the available data. Electronic reporting systems should be developed and used broadly and such applications would facilitate formal reporting among countries.

### Analysis and feedback

Meeting attendees emphasized the need to determine the incidence of severe cases through measurement of incidence rates of dengue fever, dengue hemorrhagic fever, and dengue shock syndrome, with hospitalization rates and mortality rates broken down by age group consistently applying the WHO regional case definitions. Weekly incidence of dengue, with data stratified by age, gender, and location should also be rapidly reported to allow effective use of vector control resources and to monitor intervention programs. In addition analyses should be conducted to detect and forecast dengue outbreaks through determination of the national threshold for outbreak alert and response [33], to monitor the seasonality, age distribution, and transmission patterns and to evaluate and guide the introduction of potential dengue vaccines. Vector surveillance requires baseline data for comparisons. When relevant data are available, analyses should be conducted to identify locations and patterns of the vector population (species, density, and vector-control indices) and should also be used to monitor interventions (with disease reduction as a measure of impact, and house index, container index, and Breteau index as indicators of outcome).

In conclusion, the two Dengue Prevention Boards met to discuss the practice and logistics of dengue surveillance. The attendees applied their practical experience and discussed the strengths and weakness for the countries represented at the meeting. They then suggested best practices in dengue surveillance in endemic countries. For PDVI, improved surveillance serves many purposes including generating more accurate estimates of disease burden, further demonstrating the need for a dengue vaccine, supporting clinical evaluations of candidate dengue vaccines and providing more robust surveillance for monitoring the impact of the eventual introduction of dengue vaccines in national immunization programs.

## References

[1] Gubler DJ (2002). The global emergence/resurgence of arboviral diseases as public health problems.. Arch Med Res.

[2] Beatty ME, Letson GW, Margolis HS (2009). The global burden of dengue.. http://www.pdvi.org/about_dengue/GBD.asp.

[3] Guy B, Saville M, Lang J (2010). Development of Sanofi Pasteur tetravalent dengue vaccine.

[4] World Health Organization (2005). Vaccine introduction guidelines: Adding a vaccine to a national immunization program, decision and implementation.. http://www.who.int/vaccines-documents/DocsPDF05/777_screen.pdf.

[5] Pediatric Dengue Vaccine Initiative (2009). Pediatric Dengue Vaccine Initiative.. http://www.PDVI.org.

[6] Pediatric Dengue Vaccine Initiative (2006). Dengue Prevention Boards.. http://www.dengueprevention.org/VHPB/DPB_M_page.html.

[7] DeRoeck D, Deen J, Clemens JD (2003). Policymakers' views on dengue fever/dengue haemorrhagic fever and the need for dengue vaccines in four Southeast Asian countries.. Vaccine.

[8] World Health Organization (1997). Dengue hemorrhagic fever: diagnosis, treatment, prevention and control.. http://www.who.int/csr/resources/publications/dengue/Denguepublication/en.

[9] World Health Organization (2009). Dengue guidelines for diagnosis, treatment, prevention and control.. http://whqlibdoc.who.int/publications/2009/9789241547871_eng.pdf.

[10] Southeast Asian Regional Office (1999). Southeast Asian Regional Guidelines on Dengue/DHF Prevention and Control.. http://www.searo.who.int/en/Section10/Section332/Section554.htm.

[11] Western Pacific Regional Office (2003). Western Pacific Regional Office Guidelines for Dengue Surveillance and Mosquito Control.. http://www.wpro.who.int/publications/pub_9290610689.htm.

[12] Pan American Health Organization (1997). Dengue and dengue hemorrhagic fever in the Americas.. http://www.paho.org/English/ad/dpc/cd/dengue.htm.

[13] Rigau-Pérez JG (2006). Severe dengue: the need for new case definitions.. Lancet Infect Dis.

[14] Deen JL, Harris E, Wills B, Balmaseda A, Hammond SN (2006). The WHO dengue classification and case definitions: time for a reassessment.. Lancet.

[15] Kyaw MH, Lynfield R, Schaffner W, Craig AS, Hadler J (2006). Effect of introduction of the pneumococcal conjugate vaccine on drug-resistant Streptococcus pneumoniae.. N Engl J Med.

[16] Reichert TA, Sugaya N, Fedson DS, Glezen WP, Simonsen L (2001). The Japanese experience with vaccinating schoolchildren against influenza.. N Engl J Med.

[17] Special Program for Research Training in Tropical Diseases, WHO (2004). Dengue diagnostics: proceedings of an international workshop.. http://apps.who.int/tdr/svc/publications/tdr-research-publications/dengue-diagnostics-proceedings.

[18] Krause G, Ropers G, Stark K (2005). Notifiable disease surveillance and practicing physicians.. Emerg Infect Dis.

[19] Konowitz PM, Petrossian GA, Rose DN (1984). The underreporting of disease and physicians' knowledge of reporting requirements.. Public Health Rep.

[20] BBC News (2007). Paraguay dengue official sacked.. http://news.bbc.co.uk/2/hi/americas/6422319.stm.

[21] China Times (2007). Top Tainan [China] health official sacked over dengue spread.. http://www.chinapost.com.tw/taiwan/2007/08/29/120392/Top-Tainan.htm.

[22] Daily News [Philippines] (2007). Dengue officials may be fired [Cebu, Philippines].. http://globalnation.inquirer.net/cebudailynews/news/view/20071221-108175/Dengue_officials_may_be_fired.

[23] Siang LK (2003). Worst dengue epidemic–-nation-wide campaign to demand resignation of Health Minister Chua Jui [Singapore], Democratic Action Party.. http://www.limkitsiang.com/archive/2003/jan03/lks2052.htm.

[24] Perich MJ, Davila G, Turner A, Garcia A, Nelson M (2000). Behavior of resting Aedes aegypti (Culicidae: Diptera) and its relation to ultra-low volume adulticide efficacy in Panama City, Panama.. J Med Entomol.

[25] De Paula SO, Fonseca BA (2004). Dengue: a review of the laboratory tests a clinician must know to achieve a correct diagnosis.. Braz J Infect Dis.

[26] Malcolm RL, Hanna JN, Phillips DA (1999). The timeliness of notification of clinically suspected cases of dengue imported into north Queensland.. Aust N Z J Public Health.

[27] Runge-Ranzinger S, Horstick O, Marx M, Kroeger A (2008). What does dengue disease surveillance contribute to predicting and detecting outbreaks and describing trends?. Trop Med Int Health.

[28] Ooi EE, Gubler DJ (2009). Dengue in Southeast Asia: epidemiological characteristics and strategic challenges in disease prevention.. Cad Saude Publica.

[29] Guzmán MG, Kourí G (2004). Dengue diagnosis, advances and challenges.. Int J Infect Dis.

[30] Schilling S, Ludolfs D, van An L, Schmitz H (2004). Laboratory diagnosis of primary and secondary dengue infection.. J Clin Virol.

[31] Vaughn DW, Green S, Kalayanarooj S, Innis BL, Nimmannitya S (1997). Dengue in the early febrile phase: viremia and antibody responses.. J Infect Dis.

[32] Chanama S, Anantapreecha S, A-Nuegoonpipat A, Sa-gnasang A, Kurane I (2004). Analysis of specific IgM responses in secondary dengue virus infections: levels and positive rates in comparison with primary infections.. J Clin Virol.

[33] Rigau-Pérez JG, Millard PS, Walker DR, Deseda CC, Casta-Vélez A (1999). A deviation bar chart for detecting dengue outbreaks in Puerto Rico.. Am J Public Health.

